# Quantal Basis of Secretory Granule Biogenesis and Inventory Maintenance: the Surreptitious Nano-machine Behind It

**DOI:** 10.15190/d.2014.13

**Published:** 2014-09-02

**Authors:** Ilan Hammel, Isaac Meilijson

**Affiliations:** Sackler Faculty of Medicine, Department of Pathology, Tel Aviv University, Tel Aviv 6997801, Israel; Raymond and Beverly Sackler Faculty of Exact Sciences, School of Mathematical Sciences, Department of Statistics and Operations Research, Tel Aviv University, Tel Aviv 6997801, Israel

**Keywords:** unit granule, homotypic fusion, SNARE, cellular communication

## Abstract

Proteins are molecular machines with the capacity to perform diverse physical work as response to signals from the environment. Proteins may be found as monomers or polymers, two states that represent an important subset of protein interactions and generate considerable functional diversity, leading to regulatory mechanisms closely akin to decision-making in service systems. Polymerization is not unique to proteins. Other cell compartments (e.g. secretory granules) or tissue states (e.g. miniature end plate potential) are associated with polymerization of some sort, leading to information transport. This data-processing mechanism has similarities with (and led us to the investigation of) granule homotypic polymerization kinetics. Using information theory, we demonstrate the role played by the heterogeneity induced by polymerization: granule size distribution and the stealthy machine behind granule life cycle increase system entropy, which modulates the source/receiver potential that affects communication between the cell and its environment. The granule inventory management by the same nano-machine is discussed.

## SUMMARY

1. Introduction
2. The granule alphabet
3. Secretion rate
4. Change-point recognition
5. Inventory management - tradeoff between inventory and information
6. Econo-biology of granule inventory management
7. Summary words on the surreptitious nano-machine

## 1. Introduction

Most biological systems engage in data processing. Data are transmitted by means of material movement, performed by loss of organization (*e.g.* secretory granules are degranulated, receptors aggregate) and stored by polymerization: the DNA stores information in linear sequence of four alternating basic letters; enzymes store processing information in a sequence of amino acids; receptors polymerize to transfer data into the cell. The well documented formal definition of biological language has as common basis a “string” from a list of restricted symbols, the alphabet code for DNA, RNA, proteins, etc. Another level of biological information is stored in polymers, in which the alphabet consists of a single letter, the monomer. Energy is stored as phosphate units [phosphate = the energy unit, AMP (monomer), ADP (dimer), ATP (trimer)]^1^. In neuronal transmission, miniature endplate potentials have been proved to be composed of polymers, multiples of monomers of a basic size^2-4^. Secretory granules have been demonstrated to be of quantal size (the unit granule aggregation model - Hammel, Lagunoff and Galli 2010)^5^ and single receptors are known to aggregate following binding to activator^6,7^. All of these different languages have a restricted “grammar” currently under investigation worldwide. In a number of biological systems investigated, single-letter polymers have size distributions that have been found to fit Geometric-like and Poisson-like distributions^5^, depending respectively on whether polymers of any size can expand by integration with any other polymer or growth is due to the addition of one single monomer at a time to any other polymer^5^.

**Figure 1:**
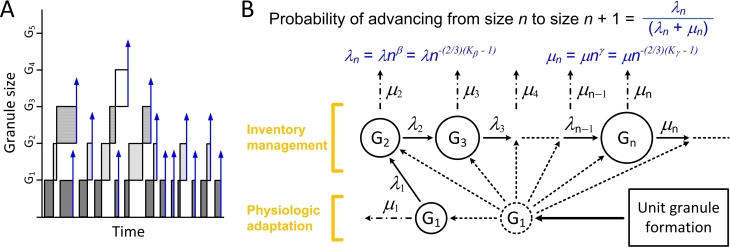
Scheme illustrating the dynamics of granule life in secretory cells (A) in which single steps in the G&E Markov model (B), based (via four global parameters *β, γ, λ, µ*) on two transition rates *λ_n_*=* λn^β^*and* µ_n_* =*µn^γ^* that describe the probability rate of a G_n_-granule (granule of quantal size n) to move one level up and become a G_n+1_-granule (*i.e.* G_n_+G_1_àG_n+1_) or to move out of the system, respectively. The transition, which occurs after an exponentially distributed time with mean *1/*(*λ_n_*+*µ_n_*), is an increase in size with probability *λ_n_/(λ_n_+µ_n_)* and exit with the complementary probability *µ_n_/( λ_n_+µ_n_).* “Birth” (Figure 1B) follows a Poisson process of formation of unit granules, each of which undergoes, independently, a Markov history as in Figure 1A. Newly formed immature secretory unit granules (broken line G_1_) can either mature (solid line G_1_) or homotypically fuse with another granule.

Our working paradigm is that immature unit granules (broken line G_1 _in Figure 1), packaged in the Golgi and released at Poisson process moments, have for some restricted time the ability to fuse with granules. Beyond this transient period these immature unit granules become (at some rate **η**) mature unit granules (solid line G_1 _in Figure 1), but some immature unit granules fuse early enough with mature unit granules (homotypic fusion), that grow as a result by unit quantal incremental size, from some size G_n_ (*i.e.*, granule of quantal size n) to the next size G_n+1_. Each mature granule is eventually secreted from the cell, by (heterotypic) fusion with the cell membrane. We have developed^8,9^ a growth and elimination (G&E) Markovian model under which mature granules grow at size-dependent rate **λ_n_**and exit the cell at size-dependent rate *μ_n_*_. _These rates are modelled by statistical mechanics reasoning so as to represent the probability of formation of the SNARE protein rosette that facilitates fusion. Letting the rosette be a ring composed of *K* SNARE complexes, *K-1* such complexes must form during some interval of time at some disk around some initial SNARE. The probability of this simultaneous event is of order

*[(area*-*disk)/(surface*-*area*-*granule)]^K-1^*=**[const_*_(area-disk)/(volume-granule)^2/3^]^K-1^=[const_*_(area-disk)/n^2/3^]^K-1^=const_*_n^-(2/3)(K-1)^.

We have accordingly modelled^9^ the growth rate as *λ_n_*= *λn^β^*and the elimination rate as* μ_n_*= *μn^γ^*, where *β=-(2/3)(K_β _-1)* and *γ=-(2/3)(K_γ _-1)* are expressed in terms of the SNARE rosette sizes *K_β_* and *K_γ_* necessary for homotypic and heterotypic fusion respectively (assumed constant and explained throughout the paper). These rates determine two natural quantal volume size distributions, the stationary distribution STAT of quantal volume of granules in the cell and the distribution EXIT of the quantal volume size of granules upon leaving the cell. These two distributions are readily evaluated from the *effective kinetics factor* *μ*/*λ*, the *effective surface factor* *γ*-*β *and the *surface tension* *γ*. **

The above applies to cells in basal mode. A cell in occasional evoked mode will secrete granules according to STAT, playing the role of the typical spatial distribution near the membrane. Thus, we expect basally secreted granules to be EXIT distributed, and evokedly secreted granules to be STAT distributed. The literature abounds with carefully measured such pairs of distributions. These are invariably multimodal, with equally spaced peaks shared by both distributions, which differ in the probabilities assigned to the common skeleton. The STAT volume is stochastically bigger than the EXIT volume. The EXIT distribution only depends on the effective kinetics and surface factors, and the STAT distribution is parametrically 2-dimensional too in essence, but the *pair* of distributions (EXIT, STAT) solidly identifies the three composite parameters above. Maximum likelihood estimation of *μ*/*λ*, *β* and *γ* was performed based on 12 such pairs of distributions^9^. The (negative) values of *β* and *γ* came out outside the range of dimensions amenable to classical analysis (van der Waals forces, etc.), but the particle physics approach applied above provides a powerful viable alternative.

The values obtained for the rosette size pairs had *K_γ_* ranging between 4 and 9 and *K_β_* usually equal to but in some cases exceeding *K_γ_* by 1. *K*-values as high as 30 have been discussed in the literature^9-11^. Theoretical calculations show that the inequality *K_γ_* ≤ *K_β_+1* is necessary and sufficient for the existence of a stationary solution to the G&E model. Entropy considerations to be described in the sequel show that the information content of the change of mode from basal to evoked is high, for given *K_β_*, if *K_γ_* is as high as possible, but the contribution of *K_γ _= K_β_+1 *over that of* K_γ _= K_β _*is not worth the instability of being borderline stable. We contend that the empirical relationship estimated between*K_β_* and *K_γ_*, under which *K_β_* is preferably equal to or exceeds *K_γ_* by 1, is just what evolution should have aimed to achieve.

**Figure 2:**
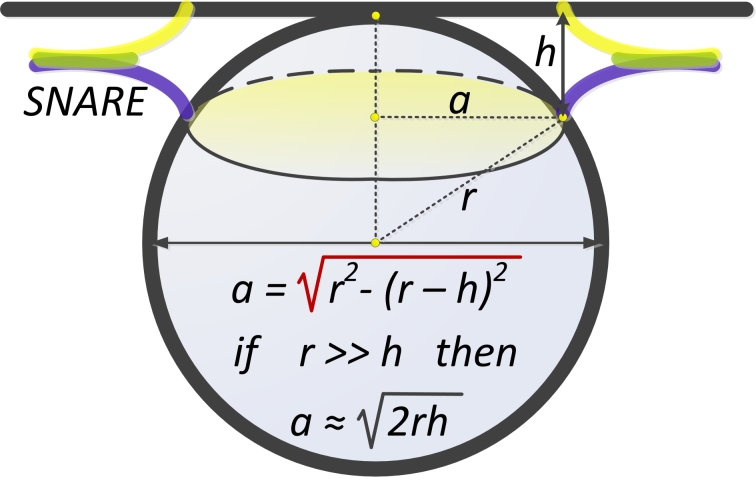
SNARE depth h is roughly constant, small with respect to granule radius (r) Hence, SNARE size *K_γ_*, proportional to the perimeter of the rosette (*2πa*), is proportional to the square root of granule diameter (*D=2r*).

The estimated values of *K_β_* and *K_γ_* are strongly increasingly correlated with granule size. The following Euclidean geometry argument supports a “square-root” rule: since SNAREs are very homogeneous, their length *h* is constant (Figure 2). Let us think of the rosette as a chordal disk in the spherical granule of radius *r*, at (the small) maximal distance *h* from the surface. Hence, the radius of this disk is **$$
\sqrt{r^2-(r-h)^2}= \sqrt{(2rh-h^2})≈\sqrt{2rh}
$$**. In other words, the number of SNARE units, proportional to the circumference of this disk, should be proportional to the *square root* of granule diameter. Linear regression of *K* on the square root of reported average diameter applied to the 12 cells in the study, led to the empirical relation $$
Kγ≈(0.9±0.2)\sqrt{D̄}
$$, ($$
D̄
$$ measured in nm).

Let **ξ** stand for the fraction of evoked secretion out of total secretion. In principle, the six parameters *η, λ, β, μ, γ* and *ξ* determine the dynamics and steady state of the entire process. We will now generalize the numerical methods to evaluate various features of the steady state of the system developed by Nitzany, Hammel and Meilijson^8^, and extend the scope of the methods to the study of transient behavior as well.

Digressing to Queuing Theory, an M/M/s queuing system is a service station with *s* servers (*s≤∞*) that serve a Poisson stream of arriving customers. Inter-arrival times are exponentially distributed with some rate *α* and service times are exponentially distributed with some rate δ, all of these times independent of each other. The basal G&E model may be viewed as a series of M/M/∞ stations in tandem, where the first station, mature monomers, has arrival rate α=η and service rate *δ=λ+μ*. A fraction *μ/(λ+μ)* of the served “customers” leave the station (secretion of mature monomers) and the remaining “customers”, dimer polymers, constitute the arrivals to the second station, with arrival rate *α=ηλ/(λ+μ)* and service rate δ=λ*2^β^+μ*2^γ^. A fraction μ*2^γ^/(λ*2^β^+μ*2^γ^) of the dimer polymers leave the station (secretion of dimers) and the remaining “customers”, trimer polymers, constitute the arrivals to the third station, with arrival rate α=η*(λ/(λ+μ))*( λ*2^β^/(λ*2^β^+μ*2^γ^)) and service rate δ=λ*3^β^+μ*3^γ^, etc. While each station reaches steady state (because the queuing system M/M/∞ is always stable), the system as a whole may not, in the sense that the steady state occupancy at the various stations may have infinite sum. As already mentioned, we have proved^8^ that global stationarity requires that *γ>β+1*.

This Markovian model lends itself to formal and/or numerical analysis, without or with the incorporation of evoked secretion, via the parameter *ξ*. The MATLAB program HM2014_mixedsecret.m admits as inputs the parameters *β, γ, μ/λ, ξ, η* and *N*, the desired expected total number of granules in the cell under steady state (all MATLAB programs are listed in the Supplementary Material, which can be accessed at https://discoveriesjournals.org/discoveries/discoveries-02-021-s001.pdf). As a first step, the program evaluates the particular values of *λ *and* μ *that achieve* N*. It then provides the stationary and exit granule quantal size distributions, as well as a menu of parameters, such as mean (stationary and exit) granule quantal size, mean and standard deviation of sojourn time in the cell, and information-theoretic *KLD *(Kullback-Leibler Divergence) measures. Although the mean *stationary* granule quantal size is considered a parameter of major interest, the product of η with the mean *exit* granule quantal size provides the production rate of unit granules at the Golgi.

There are questions of interest that defy formal or numerical analysis, about transient behavior of the G&E system. How long does it take for polymers of a give size to stabilize, and how long does it take for the total cell granule content and mean granule size to stabilize? How stable is steady state? To answer these questions as well as others the answers might raise, we have written the cell simulator Matlab program HM2014_simuLIFO.m. The name of the program is indicative of one of the salient observations, that evolutionary inventory management induces an almost Last-In First-Out discipline, where newly created monomers exit the cell soon, and occasionally created high-order polymers become trapped in the cell for longer times, as buffer for emergency needs to be filled by evoked secretion. The program can output in principle the entire history of a cell scenario for days or years, the average of 100 such scenarios, and almost any parameter the user might find of interest. The parameters are essentially provided by the output of HM2014_mixedsecret.m, with the addition of the mean batch size of evoked secretion, a parameter that does not affect steady state distributions but has some effect on the stability of steady state.

There is a good reason to study steady state stability, or fluctuations around steady state benchmark central values. As proposed, cells transmit information to the cellular environment^8^. This information, specifically about the current dichotomous mode (basal or evoked, quiescent or active neuron), is coded into the difference between the current intra-cellular and exit granule content distributions. If mean granule content *N* is large (and only then), these two distributions are most of the time close, respectively, to the (steady state) stationary and exit distributions. Besides the obvious requirement that the buffer size *N* must be large enough as backup inventory for evoked secretion, evolution must find a compromise between the need to reach steady state swiftly (by making *N* small) or maintaining stable content benchmarks (by making *N* large). It is of no wonder, then, that cells with large, few granules are only capable of supplying materials, while cells that need to impart information underwent a long process of miniaturization to achieve values of *N* in the thousands or tens of thousands, where empirical distributions typically mimic their thermodynamical limits.

## 2. The granule alphabet

The effects of changes affecting “language text” can be quantified using information theory^1^ in terms of entropy and mutual information, measures of the decrease of uncertainty at a receiver. The communication engineer asks how much information can be transmitted (or is lost) due to the loss of organization. The chemist asks for a measure of the extent to which the energy of the system is unavailable to work due to the loss of organization, and answers it by counting the number of microstates a system can occupy, to provide both a measure of the entropy of the system as well as a way of telling which processes are permissible under the Second Law of thermodynamics^1,12^. The ecologist views entropy as an index of system diversity^13,14^. The factor common to all of these definitions is that they describe ways to define the system state.

The paradigm that views polymerization as interacting monomer particles and the granule G&E life cycle as a fusion nano-machine, leads to a single-letter alphabet (the monomer) in which system state (or “word”) is polymer size. As in the best tradition of Information Theory^15^, the different distributions of secreted polymer size and secretion rates under basal and evoked secretion constitute a noisy communication channel through which the cell transmits to the environment the dichotomous information of whether it is in basal or evoked mode. Noisy channels achieve reliability of information by means of redundancy and repetition, the role played by burst size in evoked secretion.

Flory^16,17^differentiated between two major basic mechanisms of polymerization. The first is one in which a polymer of any quantal size (n) may react with any another polymer (say, of size m) to form a larger polymer (G_n_+G_m_ --> G_n+m_). Such mechanism of polymerization will lead to geometric-like distributed quantal volume in the case of non-reversible kinetics. Under such a distribution, the frequency of polymers decreases in their size, monomers being always most likely.

The alternative mechanism considered by Flory^16,17^ is the very organized and restricted process under which only a unit granule (monomer) at a time may be added to polymers of any size (G_n_ + G_1 _--> G_n+1_). Such unit addition mechanism leads to Poisson-like distributions (in reversible or non-reversible kinetics), where the most probable polymer size is close to the mean polymer size^5,16,17^. We documented in early stages of our work that granule size distribution shows a better fit to the unit addition mechanism^18,19^. Later work^8,9,11^ established the necessity to move away from classical biochemical and biophysical approaches (that assumed all monomers to be initially present and restricted granule size distributions to the two families above)^20^ in favor of a particle Physics approach under which a "packaging machine" produces unit granules of monomer size that coalesce with mature granules by the unit addition mechanism. The resulting SNARE-driven G&E model is the basis of our hypothesized mode-dependent stochastic polymer-size single-letter communication language.

The optimization process needed to construct such a language involves the imposition of constraints on polymer random frequency to detect errors efficiently^1,12^. Error detection and correction in noisy channels are based on forbidden or at least stochastically restricted letter combinations. Redundant transmission plays a role in the build-up of grammar. That such protective, redundant information accumulation has a positive survival value was pointed out by Gatlin^1^ and Dancoff and Quastler^12^. To decrease entropy and increase the fidelity of the message, the number of microstates has to be kept under proper control. Of the two polymerization mechanisms described above, the unit addition mechanism has a smaller message variety and better fidelity of message transmission than random addition, which seems to involve more redundant information content and smaller capacity, or mutual information between mode and size. Gatlin^1^ noted that the syntax of different biological languages differs in the number of letters: to increase fidelity of energy usage, energy is stored in a polymer of one type of monomer (the phosphate unit) but for the purpose of processing the huge diversity of information transferred by different ligands, a language of versatile units - proteins - is needed.

Intracellular as well as extracellular chatter face a ceaseless offensive of information. Thus, in order to elucidate the correct information the cells frequently use a single-letter language to achieve a precise and effective communication using a mechanism in which the “string message” size is regulated. In order to establish confident neural communication the unit granule size had to be decreased and made more homogeneous^8,9^. The evolutionary price tag was high turnover^8^, with an additional benefit - the rate of secretion became part of the information gain.

## 3. Secretion rate

Cells communicate with each other primarily through secretion. We have recently proposed^11,21^ that basal (or constitutive, spontaneous) and evoked (or active) secretion, that differ in calcium concentration *[Ca^2+^]*, follow comparable dependence pattern secretion rate = const1_*_E{(const2_*_[Ca^2+^])^min(X,M)^}_*_e^-K/2^ of secretion rate on *[Ca^2+^]* (with truncated stochastic number of cooperating ions *X*) and SNARE rosette size *K*. This relationship is a wide-spectrum generalization of the cell-specific *maximal* *in vitro* relationship secretion rate = const_*_[Ca^2+^]^M^ proposed by Dodge and Rahamimoff^22^, where *const* varies from cell type to cell type, and *M* is the maximal *Ca^2+ ^*cooperativity. The generalization is two-fold, encompassing all cell sizes via the dependence on rosette size *K* and all secretion modes (basal, *in-vivo* evoked, *in-vitro* evoked, etc.) via the dependence on the random number *X* of cooperating *Ca* ions, for which we applied a working paradigm making it Poisson-distributed with mean proportional to *[Ca^2+^]*. The regression coefficient of *log secretion* rate on *K* came out so close to *-0.5* that we substituted the latter, with no implied intrinsic reason. However, the order of magnitude is consistent with the G&E model, considering that *e^-K/2^=2.117^-(2/3)K^* corresponds to a representative quantal size slightly above *2*. There is at present not enough joint (*[Ca^2+^], K*)**data in the literature to estimate the value of *const1* and validate the proposed multiplicative model, but the single-variable models secretion rate = const3_*_E{(const2_*_[Ca^2+^])^min(X,M)^} and secretion rate = const4_*_ e^-K/2^ provide good fits to data. The constant *const2*, unnecessary in Dodge and Rahamimoff’s model^22^, adjusts for the volume unit chosen for recording *[Ca^2+^]*.

The first recorded evidence for slow secretion dealt with neurosecretory synapses. This type of secretion, coined minis (for miniature neurotransmission) and hypothesized as a trans-synaptic process where single synaptic vesicles spontaneously secrete from pre-synaptic neurons and induce miniature postsynaptic potentials^23^, does not induce neurons to fire and was thus considered as insignificant background noise. In contrast, basal secretion in evolutionarily older cell types has been found to be interrelated with physiological roles^24^. Thus, recent findings^23,25^of abnormal synapse development in Drosophila larvae neurosecretory cells when both basal and evoked neurotransmission are blocked, should come as no surprise: inhibiting or stimulating evoked neurotransmission alone had no effect on synaptic development, blocking minis failed to develop synapses but stimulating the secretion of more minis made synapses get bigger^25^. The information-theoretic approach introduced in the next Section, based quantitatively on KLD (Kullback-Leibler Divergence), broadens the view that the interplay between basal secretion (by itself nearly meaningless as a carrier of information) and evoked secretion (by itself an express supplier of material) constitutes the channel for efficient cellular communication.

Before embarking on a quantitative information theoretic analysis, we summarize here, and relate to the *M-K* relation, the main finding that the gradient of granule size distribution between the two modes becomes less and less informative as granules become progressively smaller, creating a need for increased differentiation of rate of secretion between the two modes. The positive dependence of secretion rate on maximal ion cooperativity *M* may be the evolutionary tool to compensate for the negative dependence on rosette size *K*. The sharp Calcium gradient between the high extracellular to the low intracellular concentration (≈0.1 μM), in the order of 10^3^-10^4^, allows for fine-tuning of evoked secretion response through Calcium gates and Calcium binding proteins, and may have evolved Calcium-triggered exocytosis of secretory granules as the main mechanism for cell-to-cell communication in animals. In the course of evolution this pattern of exocytosis has been subsequently optimized for speed: while large granules stay close to the plasma membrane in an unbound state, SNARE complexes form between small neurosecretory granules and the plasma membrane to facilitate secretion. Such bound neuro-vesicles are immediately available for secretion. Synaptotagmin, a phospholipid and Ca^2+ ^binding vesicular protein, sets the Ca^2+^ dependence of the evoked-state fusion process in neurosecretory vesicles. Synaptotagmins function as the primary Ca^2+^-sensors for most of these forms of exocytosis, acting via Ca^2+^ dependent interactions with both the fusing phospholipid membranes and the membrane fusion machinery^26^. Although many properties of the acknowledged Ca^2+^ receptor for exocytosis at the synapse have been described in a number of electrophysiological and biochemical studies, the molecular mechanisms that couple influx of Ca^2+^ and secretion of neurotransmitters have remained elusive. It is accepted, however, that fast neurosecretion involves maximal Calcium cooperativity (*M*) in the range of 4-5, as compared to *M*≤2 for slow large secretory granules: Ca^2+^ triggers various forms of exocytosis in different types of eukaryotic cells, synaptic vesicle exocytosis in all forms of neurons (3≤*M*≤5), granule exocytosis in hematopoietic cells (*M*≤2), and hormone exocytosis in endocrine cells (2≤*M*≤4) (Table 1). It is well accepted that neuro-vesicle evoked secretion is initiated by quite high Ca^2+^ concentrations within microdomains, while short-term facilitation is strongly influenced by the global buildup of "residual calcium". Neher and Sakaba^27^ documented that the intracellular Ca^2+^ concentration, at which the rate constant is 10% of the maximal rate, is in the range of 10-80 µM (initial intracellular Calcium concentration is in the order of 0.1 µM) and 1-20 µM in endocrine cells. Thus, in case of evoked state a local microdomain of [Ca^2+^] which is 4-8 times of initial concentration and *M=3* will result in evoked rate 4^3^-8^3^ (64-512).

**Table 1:** Ca2+ dependence of the rate of exocytosis in various secretory cells in correlation with morphometric data and inventory content^9,11^

Cell Type	Basal rate (per sec)	Initial maximal rate (per sec)	Granule diameter (nm)	Rosette size (K)	Granules per cell	Calcium cooperativity (M)
ANP	6	100	350	16	240	
Chromaffin cells	1.5	1500	177	11	41000	3-5
Enterochromaffin-like cells	9	160	200	12	5200	
Eosinophil	< 0.0008	0.6	500	20	400	
Juxtaglomerular cell	0.0005	0.017	1050	29	445	
Mast cell (peritoneum)	0.00024	0.8	500	20	1000	1-2
Pancreatic acinar cell	0.0033	0.62	600	22	1100	3
Pancreatic β-cell	0.01	14	305	15	11000	3-5
Parotid	0.0042	0.3-1	800	25	450	1-2
Pituitary melanotroph	0.45	25-450	160	11	20000	3
Type II alveolar cell	0.00037	0.04	1270	32	188	1-2

## 4. Change-point recognition

The receiver (receiving end in a communication channel) observes independent and identically distributed output symbols from some benchmark distribution (*e.g.*, basally secreted granule volume at a Poisson basal secretion rate **θ**), and once in a while this distribution is replaced by another (*e.g.*, evokedly secreted granule volume at a higher Poisson secretion rate θ_*_R) for some length of time. The nature of the evoked disturbance should be designed so that the receiver will detect the true advent of evoked mode with high probability, while keeping the rate of false alarms below some control limit. Clearly, this would be the case if evoked secretion rate was “infinite”, *i.e.*, if a burst of 20 granules was secreted simultaneously. Under biological feasibility constraints that bind the evoked secretion multiplier *R* to be below 10 or 50, recognition of the change should be based on all data available – secretion rate and granule size. There is a statistical theory discipline dedicated *almost* precisely to this endeavour, detecting a change-point in distribution, where once the change has happened, observations will follow *forever* the new distribution. The CUSUM statistic method (introduced by Page^28^, proved optimal by Lorden^29^ and Moustakides^30^) minimizes the expected time to detect a change after it has happened, keeping false alarm rate under control. Since in our biological application secretion reverts to the pre-change state following the evoked burst, the CUSUM expected time to detection criterion should be replaced by some longer time-window of evoked burst (say, 2SD above the mean) to render the probability of detection close enough to unity.

Let *f* and *g* stand respectively for the (known) densities of the observed data (granule volume and time since the previous secretion) before and after the change, and let *x_1_, x_2_, … x_n_* … (with *x_i_=(n_i_,t_i_)*) stand for the successive observations. The commonly used Neyman-Pearson-type statistic^31^ that best differentiates between two hypothesized stochastic regimes is the random walk

**$$
S_n=\sum_{i}^{n}{log(\frac{g(x_i)}{f(x_i)})}
$$**

whose drift is negative as long as the first regime holds but turns positive after the change. The CUSUM statistic *D_n_* is the *draw-up* at time *n, D_n_ = S_n_ – min(S_1_, S_2_,… S_n_)*, the incremental increase of the random walk from its minimal value so far. A detection is declared as soon as the draw-up exceeds some pre-assigned control limit, *i.e.*, provides enough evidence that the trend turned upwards. Some details will be provided now, to quantify detection performance and compare the CUSUM method with neuronal integrate-and-fire management. It seems indeed that evolution got advanced notice of Page’s paper^28^.

The pre-and post-change drifts of the random walk, *KLD(G,F)=Eg[log(g(X)/f(X))]* and *KLD(F,G)=-Ef[log(g(X)/f(X))]*, called Kullback-Leibler Divergence (KLD), constitute information theoretic measures of distance between the two distributions *F* and *G*. Each is the sum of two non-negative terms, the *KLD*c of secreted granule content and the *KLD*t of the (exponentially distributed) time interval from the previous secretion. It follows from the formula θ_*_exp(-θ_*_t) of the exponential density function (or rather its logarithm log(*θ*)-θ_*_t) that *KLDt(G,F)=ln(R)-1+1/R *and *KLDt(F,G)=R-1-ln(R).* These are increasing functions of the ratio *R* between evoked and basal secretion, with value obviously zero at *R=1*, a slow identical beginning *≈* *(R-1)^2^* but growing like *ln(R)* and *R* respectively thereafter. The mean number **MGD** of evokedly secreted granules until detection of this secretion mode, for a desired mean number **MGFA** of basally secreted granules until a first false alarm^8^(see also Appendix) is approximately given by MGD ≈ ln(MGFA*KLD(F,G))/KLD(G,F). For **MGFA=10^5^** (about one mistake per day at secretion rate of 1Hz), **MGD** would be over **50 **for** R=2, 10 **for** R=10, 4.5 **for** R=100 **and** 3.1 **at** R=1000 **if the STAT and EXIT distributions were indistinguishable. Since **R** has been assessed to be roughly 10 *in vivo*, an evoked burst of a few tens of undifferentiated granules would provide adequate detection. Considering that *KLDt(G,F)=1.4* for **R=10**, if the granule content contribution **KLDc(G,F)** to the denominator **KLD(G,F) **of** MGD **was of double or triple size, burst size would be cut to half or a third respectively. Table 2 displays *KLDc(G,F)* for selected cell parameters covering the cells exhibited in Section 6. It is clear that for *R>10*, an evoked burst of ten granules is borderline adequate for detection as long as the rosettes consist of at least 8 SNARE units. Miniaturization brings about the need for longer and/or faster evoked bursts. Neuro-secretory vesicles seem to require *R=100*.

The draw-up **D_n_** is stochastically smaller than the time **T_n_** it takes the random walk to reach height **n** (because at that time **D_n_** is at least **n**), somewhat easier to analyze than **D_n_**. As a premature but motivating example (developed further in the Appendix), a random walk with increments **+1** (with probability **p>1/2**) and **-1** (with probability **1-p**) will reach a positive integer threshold **n** at expected “time” (number of evokedly secreted vesicles) n/(2_*_p-1), variance n_*_4_*_p_*_(1-p)/(2_*_p-1)^3 and **CV=SD/mean** just below $$
\frac{1}{\sqrt{((2*p-1)*n)}}
$$. If **p=3/4** and expected time is **4** (see the bottom of Table 2, **R=100**) then **n=2** and **CV<1**, so a burst size of **12** (**2SD** above the mean) seems adequate. If expected time is **8** (**R=10**) then n=4, $$
CV < \frac{1}{\sqrt{2}}
$$ and the corresponding burst size would be $$
8*(1+\frac{2}{\sqrt{2}})=19
$$.

**Table 2:** Burst size needed for detection of mode change, for a wide choice of granule sizes **K_β_** – Number of SNAREs for homotypic fusion at basal state; *K_γ_* – Number of SNAREs for heterotypic fusion at basal state; *μ/λ* - effective kinetics factor; KLD_c_ - Kullback-Leibler Divergence of secreted granule content; MGS – Mean quantal granule size; MGD - Mean number of evokedly secreted granules until detection; STDGD – standard deviation of number of granules until detection; Burst – MGD+2*STDGD

Kβ	Kγ	μ/λ	MGS evoked	MGS basal	KLDC(e,b)	KLDC(b,e)	MGD (R=10)	STDGD (R=10)	Burst (R=10)	MGD (R=100)	STDGD (R=100)	Burst (R=100)
20	20	2	12.44	1.50	10.36	17.42	1.25	0.38	2.00	1.16	0.36	1.88
20	19	2	6.42	1.43	7.14	11.59	1.69	0.54	2.77	1.50	0.51	2.42
20	18	2	4.61	1.39	5.51	8.63	2.06	0.70	3.45	1.77	0.64	3.05
11	12	2	35.09	1.67	8.13	13.8	1.52	0.55	2.63	1.38	0.53	2.43
11	11	2	6.98	1.50	4.67	5.90	2.31	0.81	3.93	1.95	0.74	3.43
11	10	2	4.03	1.43	2.90	3.54	3.21	1.24	5.71	2.47	1.09	4.65
11	10	4	3.20	1.23	2.84	2.91	3.25	1.29	5.83	2.49	1.13	4.75
8	8	4	3.52	1.25	2.52	2.18	3.49	1.53	6.54	2.62	1.32	5.26
7	7	4	3.10	1.25	1.92	1.56	4.10	2.00	8.10	2.90	1.68	6.27
7	6	4	2.23	1.23	1.03	0.84	5.56	3.30	12.16	3.46	2.61	8.67
6	6	4	2.68	2.46	1.34	1.03	4.94	2.78	10.51	3.24	2.25	7.75
5	6	4	4.63	1.29	2.27	1.54	3.71	1.92	7.56	2.73	1.65	6.03
5	5	4	2.28	1.25	0.82	0.61	6.07	4.14	14.35	3.62	3.19	10.01
5	4	4	1.73	1.23	0.33	0.26	7.76	7.38	22.51	4.07	5.35	14.76
4	5	4	3.44	1.29	1.33	0.88	4.96	3.07	11.07	3.25	2.48	8.21
4	4	4	1.92	1.25	0.41	0.31	7.41	6.59	20.60	3.98	4.83	13.65
4	3	4	1.52	1.23	0.13	0.11	8.77	12.62	34.01	4.29	8.82	21.93
4	3	2	1.91	1.43	0.19	0.17	8.46	9.90	28.25	4.22	7.00	18.22

The increments of the log likelihood ratio random walk *S_n_* are linear functions of *log(granule size)* (with positive coefficient) and time interval (with negative coefficient). Hence, the monitored draw-up behaves like an action potential that decays linearly in time, is prevented from becoming negative and gets as innovation inputs the logarithm of quantal granule content. This is very similar to integrate-and-fire neuronal management modelling, where the action potential decays exponentially rather than linearly with positivity constraint. The relationship between the CUSUM statistic and integrate-and-fire neuronal management has been documented elsewhere too^32,33^. Our approach proposes the identification of action potential input innovations as logarithms of secreted granule content.

## 5. Inventory management - trade-off between inventory and information

Granule packaging, the last step following content processing within the Golgi apparatus, protects macromolecules for storage and secretion, by creating a walled compartment. Granule packaging can be viewed as a* synchronized system* of organizing macromolecules assigned for transport and secretion within the cell, a warehouse in which the granule assembles act as inventory.

Within and attached to the granule membrane there is a variety of proteins of which some, designated as package-labeling, assign the granule for correct destination^34-37^. Granule packaging by membrane enclosure and membrane labeling can be rationalized in various ways. By enclosing granule content, the packed molecules are protected from biochemical and biophysical modifications, while at the same time shielding the environment from its content. In addition, since many of the secretory molecules have hydrolytic capacity, the cell cytoplasm is also protected. Keeping such barrier contents safeguards the cell and probably increases granule content shelf life^38,39^. The cell may contain different granule types, and each such granule type may contain some molecules that cannot share the same compartment with other molecules (*e.g.* storing an enzyme with its substrate). Namely, granule packaging accomplishes a vital role in reducing the security risks of long time storage.

It is evident from Table 1 that granules contain clusters/agglomerates of a number of major molecules. In all of these cases, the number of copies of the leading molecules is in the order of 10^3^ and above. Since 1000 vesicles of unit volume will have 10 times more membrane as compared to a granule that contains the equivalent of 1000 vesicles, an increase in the macromolecule agglomerate brings about a lesser demand for content surface handling.

Granule inventory may be considered as an inventory of consumable goods, to be demanded by the environment. Shared information reduces uncertainty and reduces the need for safety stock. Thus, crosstalk between the cell and the environment must be established. The key to improved supply chain visibility, *i.e.* the capability of being readily perceived, is the sharing of information among supply chain members.

As a result, the organism becomes more responsive, and could ultimately become demand-driven rather than forecast-driven. Mason-Jones and Towill^40^ have demonstrated that “information-enriched” supply chains perform significantly better than those that do not have access to information beyond their corporate boundaries. Evolution fine-tunes the RER-Golgi pathway for packaging of goods to be supplied, to achieve agility - the ability to match supply more closely with demand. The key to agility is speed^41^ and a key characteristic of an agile organization is flexibility. If flow through the pipeline can be accelerated then unpredictable demand can be met more accurately.

Even better, less inventory makes the pipeline shorter – in effect Christopher and Towill^41^ have substituted information for inventory. The cross-talk between the cell and the environment (or the receiver) confirms knowledge of what goes on in other parts of the chain – *e.g. *granule inventory (=order status), inventory of supply for synthesis, work-in-process, pipeline inventory within the RER and the Golgi, actual demands, production plans and capacity of synthesis (=yields). These authors have established, in addition, the concept that success or failure of a supply chain is ultimately determined by the end consumer (*i.e.* the receiver). Getting the right product at the right time to the consumer "is not only the lynch pin to competitive success but also the key to survival". Uncertainty can’t be fully removed from the supply chain due to the stochastic type of product involved. For example, intrinsic properties of a pathogen that enters the tissue make demand and host survival unpredictable. Mast cell macrophage and neutrophil-specific granule inventory, confront with circumstances that require to accept uncertainty, need to develop a strategy that enables the cell to match supply and demand^38,39^. Accordingly, in many inventory systems of perishable items for consumption, the consumer controls the issuing of stock to meet demand in such a way that the movement of units through the system obeys a LIFO discipline^42^. Thus, upon pathogen invasion, the tissue activates the mast cell to secrete content upon demand.

## 6. Econo-biology of granule inventory management

Evolution has linked granule structure and function. Large granules (rosette size *K>24*) are mainly associated with adaptive tissue maintenance. The most studied cell in this class is the alveolar type-II cell of the lung (Table 1), whose main role is basal lubrication of the lung alveoli in order to reduce surface tension during breathing, about one granule per hour^43^. The next group of cells down the size ladder (rosette size *K* between *12* and *25*) is represented by the adaptive immune system (*e.g.*, mast cells, eosinophils) and the acinar cells of the gastro-intestinal system (GIT, *e.g.*, pancreatic and parotid acinar cells). These cells, which vary in their evoked secretion frequency, respond to demand dictated by the environment, whether it is exposure to an antigen (immune system)^38,39^ or to food (GIT)^44,45^. Basal secretion is minimal in the immune system to prevent tissue destruction, whereas in GIT it is about *10* times faster due to its food processing role. The third group, the hormone secreting cells (rosette size *K* between *9* and *16*), is represented by the pancreatic insulin-secreting β-cells and the chromaffin cells. These cells has well defined roles for both modes of secretion, which serve as regulators of tissues homeostatic states^46,47^.

The smallest secretory group consists of neuro-secretory vesicles (rosette size *K* between *4* and *8*), with fast-excitatory hearing and visual sensory cells at the smallest end (basal secretion rate ≈ 1 Hz) and neuro-muscular synapses at the bigger end^9,11^. We have chosen to illustrate transient and steady state inventory maintenance for cells stabilizing at a content of at least 400 granules, with rosette sizes in the vicinity of 5, 11 and 20 SNARE units.

**Figure 3:**
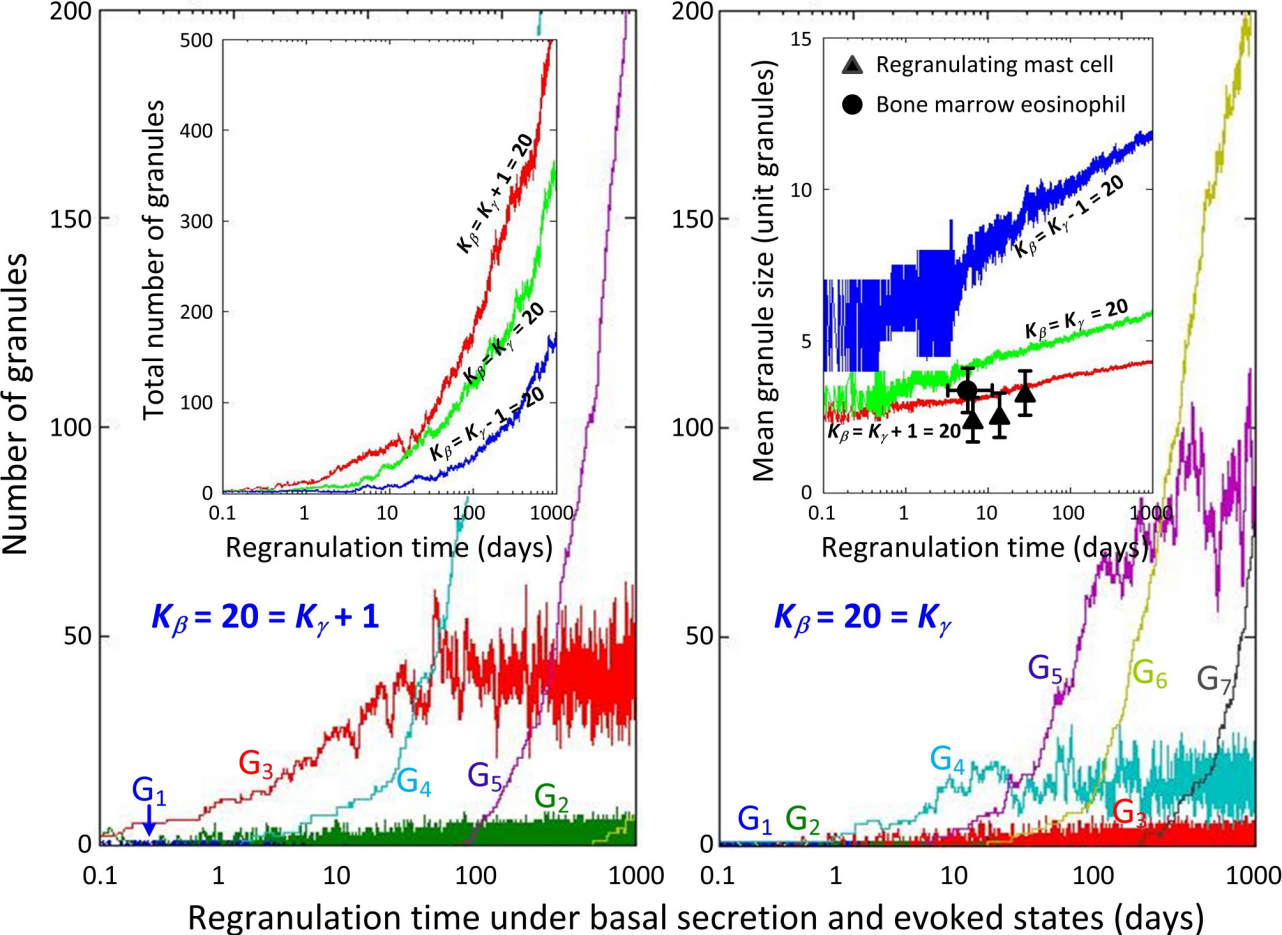
Simulations of granule inventory buildup Unit granule diameter is about 500±20 nm (*K_β_*= 20). One unit-granule (G_1_) is packed every ten minutes under the limitation of *µ*=2*λ* and heterotypic fusion of which 0.1% of the granules are secreted evokedly. Final inventory size is designed to stabilize at a total content of 1000 granules and takes significantly more than three years. Mean granule size (inset), independent of inventory size, correlates well with published data for rat mast cells^47^ eosinophil^49^ for the case of *K_β_*= *K_γ_*+1=20.

Figure 3 displays homotypic fusion rosette size *K_β_=20* and heterotypic fusion rosette size *K_γ_* between *19* and *21*, for cells with *μ=2λ* that secrete *6* granules per hour, of which *0.1%* are secreted evokedly, designed to stabilize at a total content of 1000 granules. The data demonstrates that even after 3 years the cells have not stabilized, reaching under 200 granules for *K_γ_=21* and just over 500 for *K_γ_=19* (Figure 3, inset), with mean granule size about 4 for *K_γ_=19* (consistent with Hammel *et al.’s.* experimental data^48,49^) and above 12 and still growing for *K_γ_=21* (Figure 3, inset). We see that monomers and dimers stabilize soon, trimers are the most frequent granules until stabilizing at about one month (for *K_γ_=19*), after which bigger granule polymers take over. This display supports the classical observation by Padawer^50^that granule-associated thorium dioxide remains within the cell for at least six months. These observations are consistent with mast cell and eosinophil behavior. In contrast, pancreatic acinar cells seem to compensate by an initial accelerated synthesis rate about 20 times as fast^5,51^.

**Figure 4:**
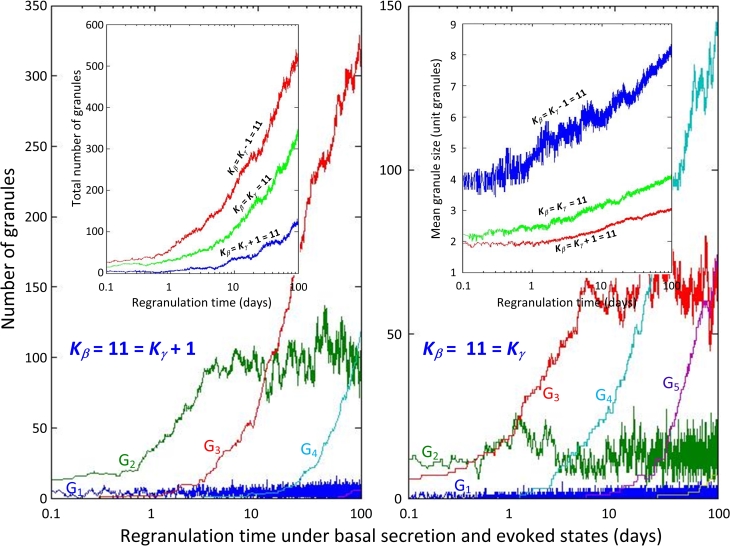
Simulations of granule inventory buildup in case of middle-sized granules Unit granule diameter is about 160±10 nm (*K*_β_= 11, with *K_γ_* differing from *K_β_* by -1, 0 and 1). One unit-granule (G_1_) is packed every ten minutes under the limitation of µ=2λ and heterotypic fusion of which 0.1% of the granules are secreted evokedly. Final inventory size is designed to stabilize at a total content of 1000 granules and takes less than a year. Mean granule size (inset), display similar pattern of growth in case *K_γ_*=10 or equal to *K_β_*.

Figure 4 displays the same parameters as Figure 3, with *K_γ_* differing from *K_β_* by -1, 0 and 1, except that *K_β_=11* instead of *20*. The process is faster, mean granule size is smaller, but the pattern is similar. Dimers are the most frequent polymer in the first day, while two weeks later bigger polymers take over.

**Figure 5:**
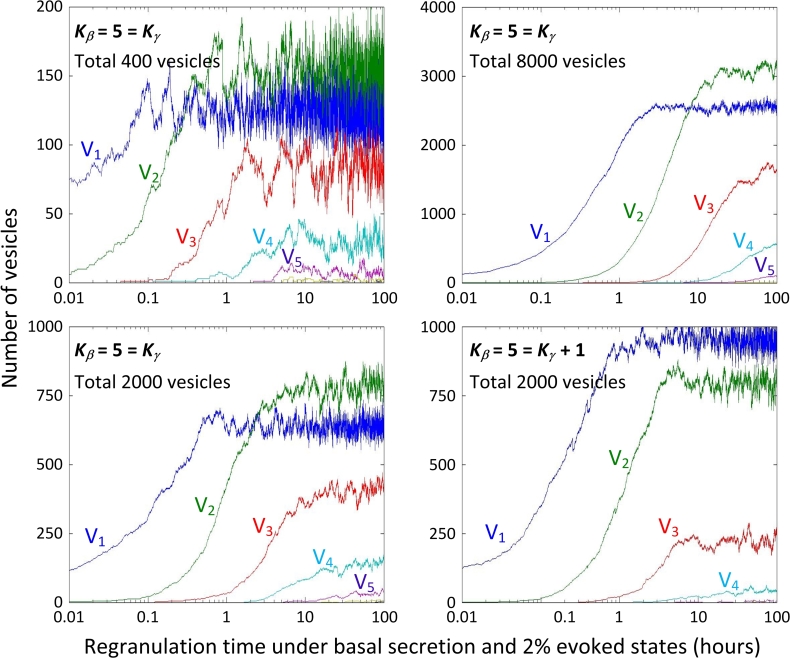
Simulations of granule inventory buildup in case of small neurosecretory vesicles Unit vesicle diameter is within the range of 35-40 nm (*K*_β_= 5, with *K_γ_* differing from *K_β_* by -1, 0 and 1, *µ=4λ*). Basal rate of secretion is 1 Hz and of which 2% of the granules are secreted evokedly. Final inventory size is designed to stabilize at a total content of 400-8000 vesicles.

**Figure 6:**
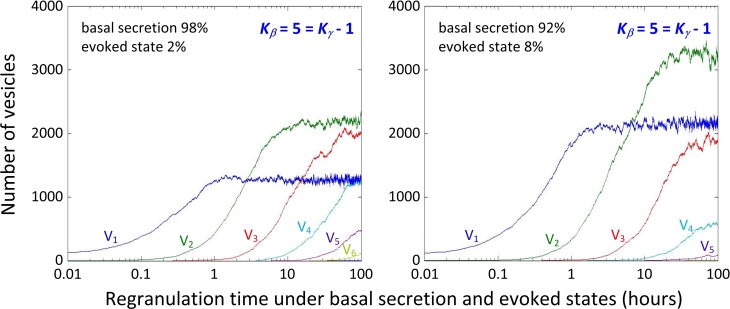
Simulations of granule inventory buildup in case of small neurosecretory vesicles. Unit vesicle diameter is within the range of 35-40 nm (*K*_β_= 5, with *K_γ_* differing from *K_β_* by 0 and 1, *µ=2λ*). Secretion rate is 1 Hz, evoked secretion constitutes 2% to 8% of total secretion, *μ=4λ* and cell content stabilizes at 2000 vesicles.

What follows illustrates a different pattern of behavior following miniaturization. Figure 5 and 6 deal with *K_β_=5* (and *K_γ_* differing by *-1, 0* and *1*). Secretion rate is 1 Hz, evoked secretion constitutes 2% to 8% of total secretion, *μ=4λ* and cell content stabilizes at 400 to 8000 vesicles. Fixing *K_β_* and *K_γ_* at *5*, we can see the effect of total granule inventory. Low content (400 vesicles) brings the cell to stabilize within a little over one hour but generates notable size fluctuations, seemingly too erratic for accurate communication. Very high content (8000 vesicles) generates very stable size distribution but pays a heavy price in terms of relaxation time, stabilizing after roughly 100 hours.

It seems that content size 2000 is a reasonable compromise. It is worthwhile to note that steady state benchmark is identical in the three pictures. Keeping granule content as 2000 but reducing *K_γ_* to 4 makes granule size stochastically smaller, with monomers becoming more frequent than dimers.

As for detection of change of mode and the impact of secretion rate magnification, by Table 2, the reduction of *K_γ_* from 5 to 4 makes mean granule size decrease from 2.28 to 1.73, while *KLD_C_(G,F)*, already as small as 0.82, decreases drastically to 0.33. However, these *KLD_C_* values are so small that granule size has only a minor impact, and detection of change of mode is primarily dictated by secretion rate amplification *R*: burst size increases from about 10 to about 12 (R=10) or from 5 to 5.4 (R=100). These figures have to be assessed in context - the last row of Table 2 shows that no granule size needs a burst size exceeding 14 (*R=10*) or 5.6 (*R=100*). In contrast, for granules with *K ≥ 7*, bursts of size 8 or smaller are adequate.

Shifting attention now to the fraction of evoked secretion, Figure 6 ascertains the minor impact of a 4-fold increase in evoked secretion on vesicle size distribution – the cell can maintain its information capacity under variable evoked demand. This complements the previous robustness finding that changes in secretion rate (equivalently, cell granule content) do not affect benchmark steady state.

An overview of the three granule size patterns displayed above shows that the proportion of monomers and dimers is very low for bigger granules and very high for smaller vesicles. As a result, turnover is high for neuro-secretory vesicles^8^, a price toll paid by miniaturization. A related heavy penalty associated with the similarity of evoked and basal vesicle size distribution brought about by miniaturization is the drastic reduction of *KLD_C_*. The contribution of size gradient to detection of change of mode is minor, and as a result the evoked burst must be bigger or faster.

The relative size of *K_β_* and *K_γ_*plays a decisive role. If *K_γ_=K_β_+1*, the cell is unstable but information-efficient. If *K_γ_=K_β_-1*, the cell is stable but less informative than it would be by either adding one SNARE unit to the *K_γ_* rosette or removing one SNARE unit from the *K_β_* rosette. The simple evolutionary strategy of learning how to build one rosette size for both types of fusion seems also the most efficient.

## 7. Summary words on the surreptitious nano-machine

The most probable main role of basal secretion is to carry out biosynthesis and secretion of recently stored cargo to the cell plasma membrane, independently of extracellular signals. In the second pathway, regulated secretion, particular secretory products are sorted and stored within secretory granules, whose fusion with the plasma membrane takes effect only if prompted by extracellular signals. These secretory proteins are frequently synthesized as inactive precursors, cleaved into their functional forms during transport from the Golgi to the immature granule and upon granule maturation^35-38^. The secretory pathway thus serves two crucial functions: (i) physiological adaptation of secretory content in response to environmental changes and (ii) granule inventory management. The core components of the heterotrimeric apparatus that mediates secretion are three SNARE proteins, VAMP, SNAP and syntaxin, that assemble to form a rosette which serves as the minimal fusion nano-machine required for fusion^52-58^. The homotypic fusion rosette size at basal state is dictated by granule size (*K_β _≈ 0.9* $$
\sqrt{D̄}
$$). Further work lead us to propose a simple equation for rate of secretion as C_*_[Ca^2+^]^ min(X,M)^_*_e^-K/2^ in which the heterotypic fusion rosette size *K* can be controlled to be within the range *1 ≤ K ≤ K_β_ +1*. Maximal evoked rate may implement K=1^8,9^ while basal secretion seems to entail *K_β_ -1 ≤ K ≤ K_β_ +1*. In this report we have integrated some mathematical basics for such stealthy machine and a description of its administration of granule inventory.

The current knowledge on the structure of individual SNARE proteins has been summarized in a number of recent reviews, which present mechanistic insights into the question of how did the fusion machine evolve into such level of complexity^52-58^. Understanding the design principles and underpinning the function of such a dynamic modular protein system is a challenging task. Characterization of rate of secretion, a fundamental problem since the 1950s work of Sir Bernard Katz^4^, has been investigated using classical biophysics models or curve fitting to general equations (*e.g.* the Logistic curve, Hill plot *etc.*). The main drawback of these smooth approaches is that these rules "obey well" within a close range of data, for restricted cases. In a recent manuscript^9^ we proposed an alternative statistical-mechanics-based granule lifecycle G&E model. The current manuscript brings forward the wide-spectrum secretion rate relation C*[Ca^2+^]^min(X,M)^ *e^-K/2^ as a facet of the mathematical G&E model^11^.

The validity of the statistical mechanics approach, that covers all biological range of [Ca^2+^], secretion rates (10^-5^–10^5^ granules per millisecond)^11^ and granule dimensions, has been checked on 27 different cells, with granule volume range 8x10^3^–1.1x10^8^ nm^3^. This *M-K* relation is parsimoniously based on only two or three physical parameters: ion maximal (*M*) and actual (X) cooperativity, and the number of SNARE units (*K*) needed for fusion. We are aware of no earlier attempt to try on this subject discrete statistical mechanics approaches; classical biophysics and continuous space formulas sound more "scientific" than controversial probability assessments. However, within the small volume in which fusion reactions occur (≈1.4x10^-26^ m^3^), classical biophysics probably plays a minor contribution.

A significant part of this review dealt with granule inventory management and its evolution to the establishment of a simple communication machine. It emerges that such stealthy machine can manage granule hierarchy since rate (dictated by *K*) is highly correlated with granule size. While most cell types possess an inventory of granules of a single type, some secretory cells have a heterogeneous arsenal of granules of different type/content. Evidently, the emergence of a simple SNARE nano-machine that must manage secretion dictated by just very few parameters (the *M-K* relation) is a challenging evolutionary task. Since all granules "perceive" upon activation similar calcium concentration^59^, the cell’s option to monitor granule hierarchy is limited to the management of granule inventory by assigning to each granule type a rosette *K*-size. Namely, granule hierarchy is dictated by granule size^11^. Since secretion rate is linearly correlated with *e^-K/2^*, increasing *K* by four SNARE units leads to ≈7.4X acceleration of the secretion rate. However, since granule diameter is linearly correlated with *K^2^*, the price for a *∆K=4* increase in rosette SNARE size means a *(K+4)^2^/K^2^X* increase in granule diameter. In case *K*=8, 12, 16 and 20, granule diameter will increase (due to *∆K=4*) by a factor of 2.25, 1.78, 1.56 and 1.44 respectively. There are two cells which were identified to have at least three granule types, polymorphonuclear neutrophil and platelets. Polymorphonuclear neutrophil (PMN) has two oblate *D_1_*≈85x209nm (*K*≈8-12), *D_2_*≈124x305nm (*K*≈9-15), and one spherical *D_3_*≈260nm (*K*≈14) granules^60-62^. Platelets have *D_1_*≈150nm (*K*≈11), *D_2_*≈200-250nm (*K*≈12-14) and *D_3_*≈200-400nm (*K*≈12-18)^63-65^. Both cells store active enzymes in the large granules and their substrates in the smallest granules. Thus, small granules will be queued first for secretion and the largest granules will be last. As such, the active enzymes will mainly process the substrates with less chances for tissue destruction. To decrease basal secretion of harmful proteins the PMN cells terminate granule synthesis (increase *λ *and thus decrease* µ/λ*) and generate ellipsoid-like granules (and thus increase *K*).

The co-existence of two secretory pathways within the same cell, basal and evoked, requires correct categorization of secretory content and vesicle machinery to each pathway^66^. Our model suggests that there may be no need for specialized sorting of cargo to each pathway: the cell selectively dictates synthesis of the new set of proteins to be secreted in response to environmental demands. The newly synthesized content is assigned to the newly formed granules and gets priority for secretion^8,9^. Newly formed granules which are not secreted, are incorporated to the granule inventory according to the stochastic pathway in Figure 1. Thus, the evolutionarily generated secretion machinery will assign mature granules to be secreted in the regulated pathway mostly upon stimulation.

We hope and predict that the statistical mechanics approach applied herein, in which a single simple machine emerges as a multitasking nano-machine, will have a key changing effect on the investigation of the pathophysiology of secretory mechanisms and on biophysical methodology for the investigation of secretion, beyond the electrophysiological, amperometric and fluorescence measurement approaches applied so far.

## Open Questions

1. Which SNARE proteins dictate secretory granule pools with different secretion probabilities?
2. Since maturation of immature granules is under intracellular control and is integrated into higher regulatory networks, what are the proteins that dictate it?
3. Is there a unifying control mechanism for fusion?
4. What is the biophysical meaning of the effective kinetics factor μ/λ?
